# Transcranial Alternating Current Stimulation (tACS) as a Tool to Modulate P300 Amplitude in Attention Deficit Hyperactivity Disorder (ADHD): Preliminary Findings

**DOI:** 10.1007/s10548-020-00752-x

**Published:** 2020-01-23

**Authors:** Isa Dallmer-Zerbe, Fabian Popp, Alexandra Philomena Lam, Alexandra Philipsen, Christoph Siegfried Herrmann

**Affiliations:** 1 Department of Complex Systems, Institute of Computer Science, Czech Academy of Sciences, Prague, Czech Republic; 2 Department of Physiology, Second Faculty of Medicine, Charles University, Prague, Czech Republic; 3 Experimental Psychology Lab, Department of Psychology, European Medical School, Cluster for Excellence “Hearing for All”, Carl Von Ossietzky University Oldenburg, Ammerländer Heerstr. 114-118, 26129 Oldenburg, Germany; 4 Research Center Neurosensory Science, Carl Von Ossietzky University, Oldenburg, Germany; 5 Department of Psychiatry and Psychotherapy, University of Bonn, Bonn, Germany; 6 Medical Campus University of Oldenburg, School of Medicine and Health Sciences, Psychiatry and Psychotherapy – University Hospital, Karl-Jaspers-Klinik, Bad Zwischenahn, Germany

**Keywords:** P300, Attention deficit/hyperactivity disorder, Transcranial alternating current stimulation

## Abstract

Studies examining event-related potentials (ERP) in patients affected by attention deficit/hyperactivity disorder (ADHD) have found considerable evidence of reduced target P300 amplitude across different perceptual modalities. P300 amplitude has been related to attention-driven context comparison and resource allocation processes. Altered P300 amplitude in ADHD can be reasonably assumed to be related to ADHD typical cognitive performance deficits. Transcranial alternating current stimulation (tACS) can increase the amplitude of endogenous brain oscillations**.** Because ERP components can be viewed as event-related oscillations (EROs), with P300 translating into the delta (0–4 Hz) and theta (4–8 Hz) frequency range, an increase of delta and theta ERO amplitudes by tACS should result in an increase of P300 amplitudes in ADHD patients. In this pilot study, 18 adult ADHD patients (7 female) performed three consecutive blocks of a visual oddball task while the electroencephalogram (EEG) was recorded. Patients received either 20 min of tACS or sham stimulation at a stimulation intensity of 1 mA. Individual stimulation frequency was determined using a time–frequency decomposition of the P300. Our preliminary results demonstrate a significant increase in P300 amplitude in the stimulation group which was accompanied by a decrease in omission errors pre-to-post tACS. However, studies including larger sample sizes are advised.

**Electronic supplementary material:**

The online version of this article (10.1007/s10548-020-00752-x) contains supplementary material, which is available to authorized users.

## Introduction

ADHD patients have been consistently reported to show a reduction in P300 amplitude as compared to healthy controls. This alteration of P300 was obtained in ADHD children (Gow et al. 2012; Johnstone and Barry 1996; Senderecka et al. 2012; Strandburg et al. 1996; Tsai et al. 2012), as well as in ADHD adults (Grane et al. 2016; Hasler et al. 2016; Itagaki et al. 2011; Woltering et al. 2013; see Szuromi et al. 2011 for a meta-analysis), while patients were performing oddball and similar cognitive tasks.

In terms of ADHD treatment, pharmacological treatment with stimulants is the treatment of first choice (Ebert et al. 2003). However, 20–75% of adults with ADHD do not respond sufficiently to medical treatment (Wilens et al. 2012) and the discontinuation rate is high (Zetterqvist et al. 2013). In this context, non-invasive brain stimulation is an attractive treatment alternative because of its safety profile (Antal et al. 2017) and flexibility (Demirtas-Tatlidede et al. 2013). While reviews on non-invasive brain stimulation in the context of ADHD treatment stress the advantages of transcranial electrical stimulation, they conclude that further research on its application in ADHD is needed (Demirtas-Tatlidede et al. 2013; Palm et al. 2016; Rubio et al. 2016). The potential of transcranial alternating current stimulation (tACS) in the treatment of ADHD has not been explored so far. Furthermore, just like all generally accepted treatment approaches for ADHD, the few existing studies on transcranial direct current stimulation (tDCS) effects in ADHD did not target P300 deficits (Bandeira et al. 2016; Breitling et al. 2016; Cosmo et al. 2015; Munz et al. 2015; Prehn-Kristensen et al. 2014; Soff et al. 2017; Soltaninejad et al. 2015). Even though P300 has not been in the focus of treatment interventions for ADHD, its manipulation by ADHD treatment has been reported and been linked to the improvement of ADHD symptomatology (Jonkman et al. 2007; Paul-Jordanov et al. 2010; Schoenberg et al. 2014; Zillessen et al. 2001).

Like tDCS, tACS represents a form of transcranial electrical stimulation with weak electrical currents of approximately 1–2 mA applied through two or more electrodes attached to the scalp. Unlike tDCS, which imposes a direct current, the applied current in tACS alternates at a certain frequency (Herrmann et al. 2013). Due to its sinusoidal current, tACS has been demonstrated to modulate endogenous brain oscillations (e.g., Helfrich et al. 2014).

### P300 Amplitude and Behavior

P300 (or P3b) generation has been associated with processes in later stimulus processing stages (as compared to the earlier occurring P3a component), when targets need to be discriminated from standards for response selection. These attention-driven processes involve working memory, when stimuli are compared against the remembered mental schemes of the (non-) targets which in turn will be updated if necessary (Polich 2007). This widely accepted model is called the *context updating theory* of P300 (Donchin and Coles 1988; Linden 2005; Polich 2003). While P300 latency is understood to reflect processing speed, P300 amplitude is related to the intensity of processing (Kok 2001). Because P300 amplitude has been shown to depend on stimulus probability (with amplitude and probability being negatively correlated) and task difficulty, it has been connected to attention-driven resource allocation processes (Polich 2007). In this framework, the ADHD-related reduction of P300 amplitude is attributed to a mal-functioning of resource allocation and can be related to ADHD typical cognitive performance deficits that are observed in visual oddball/go-nogo tasks. These comprise increases in reaction time mean and reaction time variability, a well as higher error rates for false alarms (commission errors), and omission errors (Bezdjian et al. 2009; Derefinko et al. 2008; Hervey et al. 2004; Uebel et al. 2010). Among these findings, reaction time variability is probably the most commonly reported one (Tamm et al. 2012). The assumption of a relationship between P300 alterations and ADHD symptomatology seems likely, given the obvious attention-related nature of its functional correlates. The relationship has been supported by correlational (Woltering et al. 2013) as well as other studies investigating treatment interventions showing effects on P300 amplitude: Schoenberg et al. (2014) showed a normalizing effect of Mindfulness-Based Cognitive Therapy on P300 alterations in ADHD. An increase in P300 amplitude was accompanied by an improvement in symptomatology. Further, an ameliorating effect of ADHD medication on error rates while showing the inverse effect on P300 amplitude was found (Jonkman et al. 2007). From this likely relationship it can be followed that when P300 characteristics are changed, related deficits should change as well. In addition to the relational argument, tACS has been proven to be able to influence cognitive performance and to decrease reaction times (Antal et al. 2008; Antonenko et al. 2016; Fröhlich et al. 2015; Kar and Krekelberg 2014; Polanía et al. 2012).

### Delta/Theta Activity

**Fig. 1:**
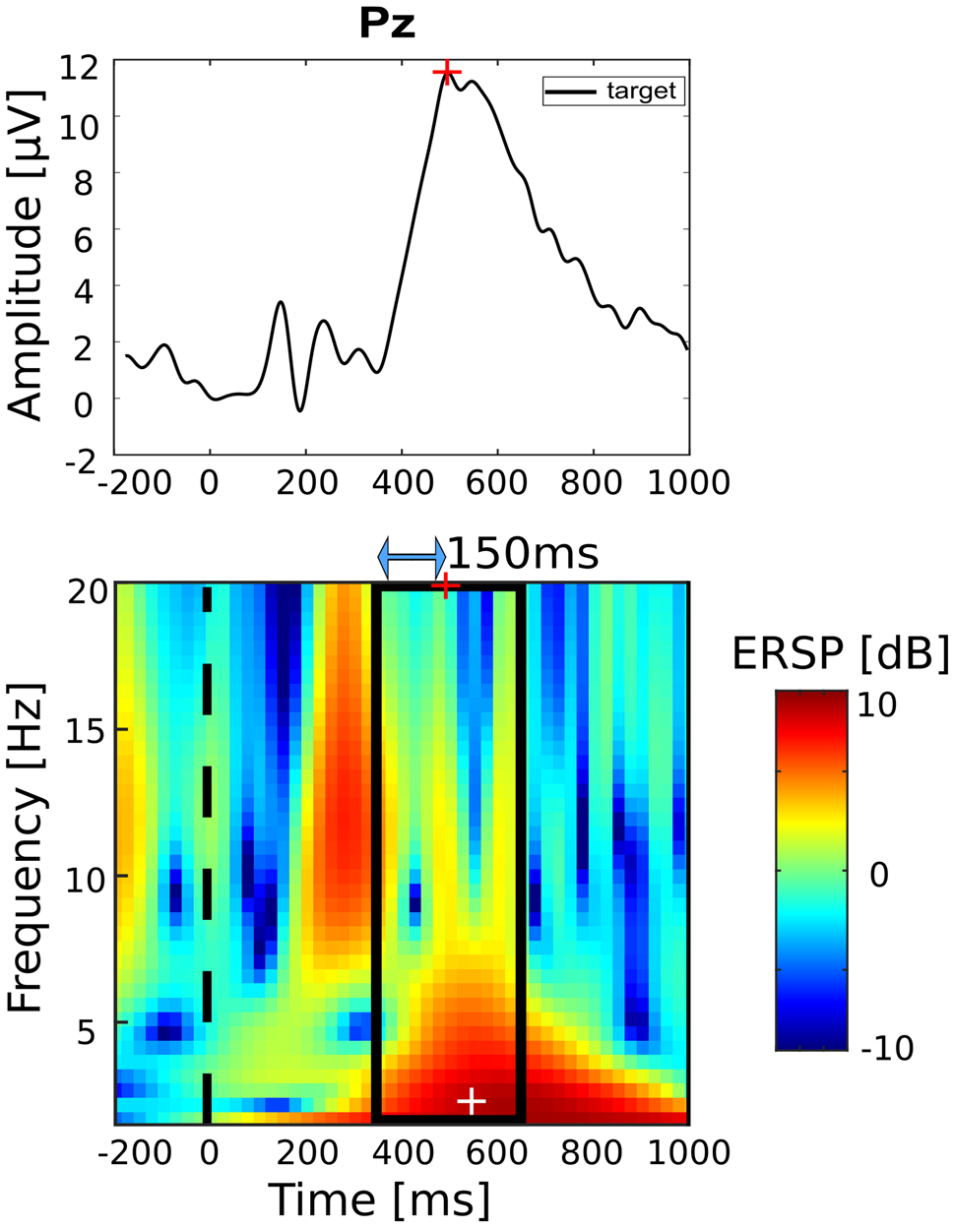
P300 in the time (top) and time–frequency domain (bottom). Stimulation parameter estimation: after P300 latency estimation (Pz maximum between 300 and 600 ms after stimulus onset; marked by red cross), ERSP maximum (white cross) is found in ± 150 ms window (marked by black rectangle) around P300 latency. P300 latency used for setup of stimulation timing, frequency at ERSP maximum used as stimulation frequency

ERPs components like the P300 are conceptualized as brief and transient brain responses to an occurring event. Brain oscillations, that are typically targeted by tACS interventions are usually associated with longer durations. When conceiving ERPs as part of oscillations like that, they can be viewed as event-related oscillations (EROs; Başar 1998; Herrmann et al. 2014). Another aspect why ERPs and EROs are classically dealt with separately is that ERPs are typically viewed in the time domain only, while EROs are represented in the time–frequency domain. However, ERPs also can be investigated in the time–frequency domain, which has even been demonstrated to offer valuable insight in the context of their functional cognitive correlations (Başar et al. 1999). Figure 1 displays an exemplary ERP wave represented in the time (upper panel) and in time–frequency (lower panel) domain, taken from this study. Later ERP components like the P300 are captured in lower frequency ranges, namely theta (4–8 Hz) and delta (0–4 Hz) range. Based on these considerations, we assume that tACS could potentially influence ERPs in the same way as it can influence brain oscillations in general.

When viewing the P300 as part of an oscillation or looking at it in the time–frequency domain, it is represented by an ERO in the delta/theta frequency range. In line with that view, several studies were able to illustrate the functional link between oscillatory activity in the delta and theta frequency range and the generation of the P300 complex in auditory and visual oddball tasks (see the extensive review of Güntekin and Başar 2016). First, a frequency specific power increase was demonstrated for the delta and theta frequency range in the target compared to the non-target condition (Başar-Eroglu et al. 1992, 2001; Demiralp et al. 2001; Kolev et al. 1997; Schürmann et al. 1995). Second, time–frequency components in the delta and theta frequency range occurring at the typical latency of the P300 component were identified even on a single trial basis and matched the characteristic parietal scalp topography of the P300 (Demiralp et al. 1999, 2001; Kolev et al. 1997; Quian Quiroga et al. 2001). Consequently, delta and theta oscillations represent a possible mechanism for the generation of the P300 component.

tACS has been shown to increase the amplitude of endogenous brain oscillations within the stimulated frequency range during the time of stimulation the so-called on-line effect of tACS (Helfrich et al. 2014; Neuling et al. 2015). In addition, tACS results in elevated EEG amplitudes at the frequency of stimulation also after the end of stimulation. This effect is referred to as off-line effect or after-effect (Neuling et al. 2013; Zaehle et al. 2010). For the alpha frequency range, this off-line effect lasts for at least 30 to 70 min after stimulation (Kasten et al. 2016; Neuling et al. 2013; Zaehle et al. 2010). The on-line effects are often explained by entrainment, i.e., by EEG oscillations synchronizing to a rhythmic external force or spike-timing dependent plasticity (Thut et al. 2011; Vossen et al. 2015). Recent research demonstrated that tACS is capable of influencing brain oscillations. As reviewed above, a functional link of oscillations in the delta and theta frequency range with the P300 component has been shown. Consequently, targeting this ERP component using tACS represents a promising approach of modulating the P300 response.

In summary, the present study evaluates the potential of tACS to modulate the altered P300 amplitude in ADHD. The key points made so far are: a reduction in P300 amplitude in ADHD patients compared to healthy controls has been reported. This amplitude reduction is understood to reflect difficulties in attention resource allocation processes and a link between P300 and ADHD typical symptomatology has been shown. Furthermore, it has been demonstrated that ERP components can be viewed as EROs. Therefore, tACS can be assumed to influence ERPs in a similar way as it influences brain oscillations in general. The P300 component’s oscillatory equivalent is an ERO in the delta/theta frequency spectrum. Hence, it can be targeted with tACS stimulation within respective frequency range. tACS of a certain frequency can increase the amplitude of brain oscillations within the respective frequency band. Therefore, P300 amplitudes should be increased via tACS stimulation. Based on these considerations, the following directed hypotheses were formulated:

- Hypothesis 1: P300 amplitude modulation. When comparing two ADHD patient groups receiving either delta/theta tACS or sham stimulation, patients in the stimulation group will show an increase in P300 amplitude pre-to-post stimulation while patients in the sham group will not show the same effect.
- Hypothesis 2: behavioral effects. Along with the hypothesized increase of P300 amplitude, we hypothesize that tACS will induce a reduction of ADHD-related cognitive performance deficits, i.e. a decrease in mean reaction time and of reaction time variability as well as in omission and commission type error rates.

## Materials and Methods

### General Study Design and Equipment

The present study is a sham-controlled study on individuals diagnosed with ADHD. The study is designed as a two × two mixed design with factors *time* (pre/post intervention) and *group* (stim/sham). Each patient attended one experimental session of approximately two and a half hours duration. The session was split into three blocks (pre, during, post conditions), the first and last of which were EEG-only blocks. In the second block, tACS stimulation or sham stimulation was applied. Measurements took place at the laboratories of the Experimental Psychology Lab in the Department of Psychology at the University of Oldenburg, Germany, in an electrically shielded room. A 32 channel EEG was recorded according to the international 10–10 System plus right Electrooculogram (EOG). Signals were amplified using a BrainAmp amplifier (Brain Products, Gilching, Germany), digitized at a sampling rate of 1000 Hz. The amplitude digitization range of the amplifier was ± 3.2768 mV with a resolution of 0.1 µV. tACS was delivered using a battery-operated stimulator system (DC-stimulator plus, Neuroconn, Ilmenau, Germany). The visual task was implemented using Presentation software (Version 18.01, Neurobehavioral Systems Inc., Albany, CA, USA). Data preprocessing and outcome variable extraction were conducted with Matlab (Version 9.2.0, The Mathworks Inc, Natick, MA, USA) and the interactive Matlab toolbox eeglab (Delorme and Makeig 2004). Statistical analyses were conducted with Matlab and the R software package (Version 3.3.0, R Foundation for Statistical Computing, Vienna Austria).

### Study Sample

The study sample comprised 18 patients (7 females, age: *M* = 31.3, *SD* = 9.89 years, ranging from 19 to 57 years of age). Nine patients received tACS stimulation, the other nine received sham stimulation The reader is referred to Table S2 (Online Resource) for all sample characteristics specified by group. All patients were diagnosed with ADHD and were currently undergoing treatment at the specialized outpatient clinic for adult ADHD of the University Hospital for Psychiatry and Psychotherapy at the University Oldenburg, Germany. Diagnosis according to DSM-IV was based on psychiatric expert assessment and was validated using observer rating scales and self-rating scales including the Wender Utah Rating Scale (Retz-Junginger et al. 2002), the ADHD diagnostic checklist (Rösler et al. 2004), the Structured Clinical Interview for DSM-IV Axis I Disorders (SCID-I) and Structured Clinical Interview for DSM-IV Axis II Personality Disorders (SCID-II) (Fydrich et al. 1997; Wittchen and Fydrich 1997). Concurrent use of ADHD medication (reported by nine of the participating patients; agent: methylphenidate) was discontinued at least three days prior to the measurement under the supervision of the medicating doctor. Exclusion criteria comprised left-handedness, metal near brain or skull, epilepsy in medical record or direct family, comorbid neurologic conditions, severe affective disorders and schizophrenia, intake of psychopharmacological medication, substance addiction, autism, dermatosis and pregnancy. Participation was voluntary. Eligible patients, as decided by their medicating doctor, would be contacted by phone and invited to take part, if inclusion criteria were met. Patients received monetary compensation. Written informed consent was obtained from all participants. All investigations were in accordance with the Declaration of Helsinki and the GCP (good clinical practice). The study received ethics committee approval from the medical ethics review committee of the University of Oldenburg.

### Procedure and Task

**Fig. 2:**
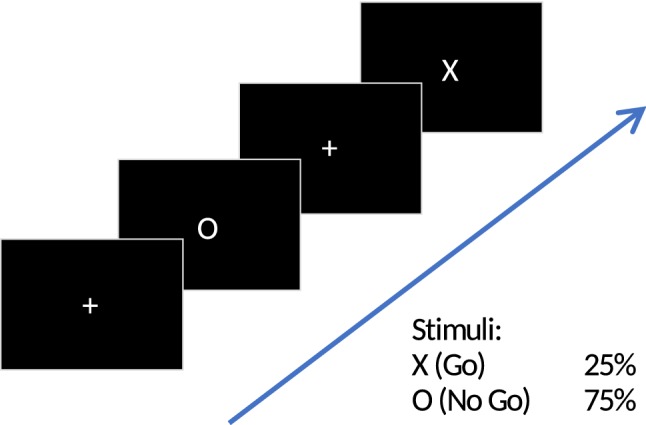
Visual oddball task

Eligible patients were randomly allocated to groups in a one-to-one ratio using a simple randomization script. The experiment consisted of three blocks (pre, during, post) during which the patients sat in a dark room in front of a computer screen performing a visual oddball task. The task consisted of frequent, irrelevant ‘O’-stimuli (0.75 probability of occurrence), further referred to as standards, and infrequent ‘X’-stimuli (0.25 probability of occurrence), further referred to as targets. Both letters were presented in white on black background (see Fig. 2 for schematic representation). In response to the targets, a button was to be pressed with the right-hand index finger. In between stimuli, the patient was instructed to fixate on a cross symbol displayed in the center of the screen. Stimuli were presented for 1000 ms, inter-stimulus interval (fixation cross displayed) jittered between 1000 and 2000 ms. The pre and post condition included 400 trials (approximately 300 standards and 100 targets) with an average duration of 16.6 min (400 × 2500 ms). The during condition had a fixed duration of 20 min, after which stimulator and visual presentation turned were off automatically. Stimulus size was 7 mm × 7 mm for fixation crosses, and 10 mm × 10 mm for targets and standards. Patients’ distance from the monitor was 1 m.

### tACS Configuration and Parameters

In the stim condition 20 min of tACS stimulation were delivered at an intensity of 1 mA (peak-to-peak). 20 min stimulation length was chosen as it was the maximum stimulation length approved by the local ethics committee at that time. The current was ramped up and down over the first and last 10 s of stimulation in order to minimize discomfort. In the sham condition, the procedure and stimulation parameters (see below) were set and determined in the same way as for the stim condition to blind the patient from the experimental condition. In contrast to the stim condition, the stimulation in the sham condition faded in for 10 s and after reaching the amplitude of 1 mA, the signal immediately faded out for another 10 s. All stimulation parameters were manually entered to the device. Therefore, the experimenter could no longer be blinded after the pre condition ended. EEG electrodes were used for stimulation. Before running the stimulation, chosen EEG electrodes were connected to the stimulator (further details below). Patients were then familiarized with the stimulation. The waveform of the applied stimulation was sinusoidal without DC offset.

There are two fundamental aspects that we considered requirements to the successful manipulation of P300 via tACS stimulation: for one, the phase of the oscillatory stimulation current and the phase of the theoretical P300 oscillation should match. Otherwise, stimulation peaks could coincide with P300 oscillation troughs, which would result in cancelling out the P300 wave instead of amplifying it. Secondly, the electrode montage should be designed to direct the current towards the areas of the brain involved in P300 generation.

To meet these requirements, stimulation parameters were determined on a single patient basis. After the pre condition ended, data were briefly preprocessed (low-pass filter: 20 Hz, high-pass filter: 0.5, epoch length: − 3 to 4 s around stimulus onset, baseline removal relative to − 50 to 0 ms interval). In the following, mean P300 latency (in ms, relative to stimulus onset) was estimated. This was done by finding the maximum value of the averaged ERP wave at electrode Pz between 0 and 900 ms after stimulus onset. Visual oddball tasks have a typical P300 latency of 400 ms (Polich and Criado 2006). Accordingly, mean amplitude latency in a pilot study including healthy subjects was 440 ms (Popp et al. 2019). Figure 1 illustrates stimulation frequency estimation. A time–frequency decomposition was computed using a wavelet transform (epoched Pz data: − 3 to 4 s normalized to − 3 to 0 s baseline interval, frequency range: 1.5 to 20 Hz, 3 cycles in each analysis wavelet, frequency resolution: 0.5 Hz, time resolution: 0.024 s). Individual stimulation frequency was the frequency at the maximum event-related spectral perturbation (ERSP) within ± 150 ms time window around individual P300 latency (maximum in time of maxima in frequency). The employed rationale was that P300 ERO would be reflected by the strongest contributing frequency component within the P300 time window (see “Introduction” section). The time interval around P300 latency was introduced in order to account for temporal smearing of the employed time–frequency decomposition method. Resulting stimulation frequencies had a mean of 3.00 Hz and a standard deviation of 1.24 Hz (*M*_*stim*_ = 3.06 Hz, *SD*_*Stim*_ = 1.40 Hz).

#### Stimulation Timing

**Fig. 3:**
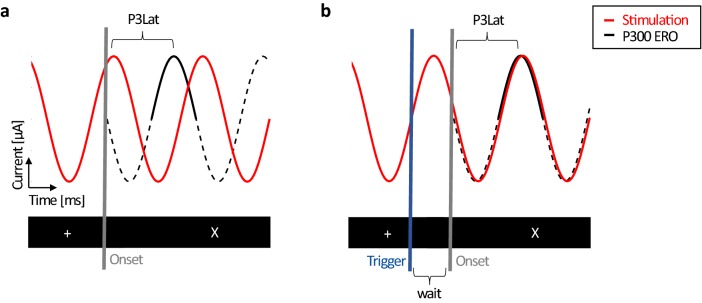
Stimulation timing setup. **a** Stimulation (red) and P300 (black) sinusoids on top, visual presentation depicted underneath (black bar). **b** Presentation is programmed to wait until stimulation phase and P300 ERO phase match before switching from fixation cross ‘ + ’ to stimulus ‘O’/’X’ presentation. Wait duration was equal to the calculated time between stimulation peak and P300 peak latency (P3Lat)

In order to tailor the stimulation individually, estimated P300 latency and ERO frequency were used to manipulate the timing of stimulation. To ensure that P300 latency would coincide with a stimulation peak, the general presentation script was individually adapted as follows. At every zero crossing of the tACS stimulation (at the beginning of each oscillatory cycle) the stimulator sent a trigger to the presentation computer. Before a visual stimulus would be presented on the screen, a wait-trial (wait) was inserted that covered the time until P300 latency and the stimulation peak of tACS would co-occur (see Fig. 3). Since stimulation duration was set to 20 min, this procedure resulted in different numbers of stimulus trials in the during condition (more trials for higher stimulation frequency; resulting mean trial number of 418.7 ± 32.3). To monitor stimulus onset timing throughout the session, a diode channel was attached to the presentation screen. In this way, errors in the script that could have caused undesired time jitters were ruled out.

#### Electrode Montage

**Fig. 4:**
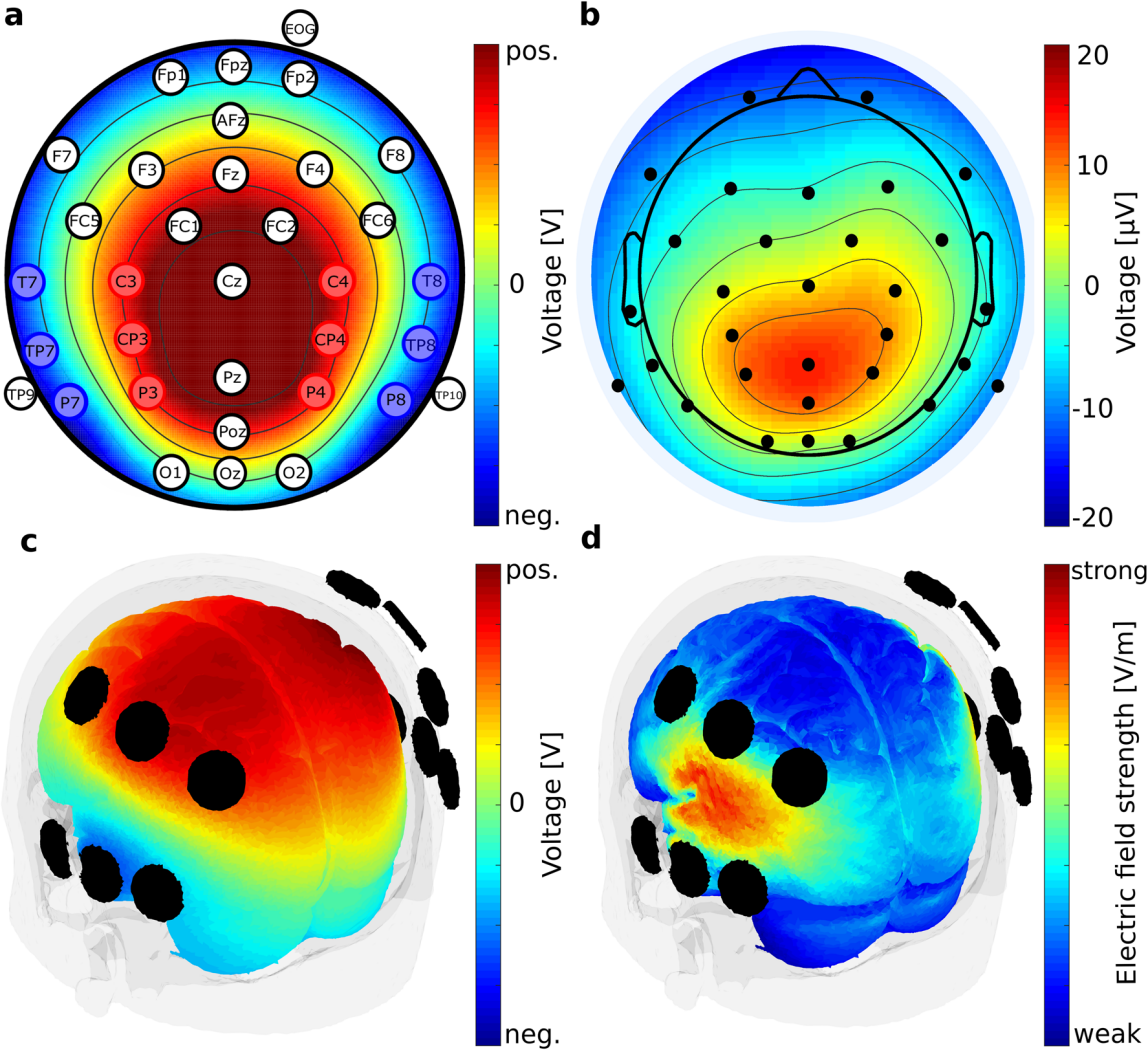
Stimulation electrode montage. **a** Electrode positions of the EEG electrodes according to the international 10–10 system. Red and blue positions indicate electrodes used for tACS in the during condition of the experiment. Electrodes used for tACS are arranged in two electrode clusters, red and blue. Each cluster is used as one tACS electrode (alternatingly anode or cathode). Color map shows voltage topography on the scalp resulting from the two clusters of stimulation electrodes. Depicted is the case of the medial cluster being the anode and the lateral cluster being the cathode. tACS amplitude was set to 1 mA peak-to-peak. Voltage was adjusted automatically by the stimulation device according to individual electrode impedances. **b** P300 component topography of the EEG baseline condition, averaged over subjects at the subject’s individual P300 peak latency. **c** Voltage topography distribution on brain surface obtained from finite element modelling using the mentioned stimulation electrode clusters and a current strength of 1 mA peak-to-peak. Modeling was performed using the ROAST toolbox (Huang et al. 2017) **d** Electric field distribution on brain surface obtained from finite element modelling with the same parameters as in c. Figure and caption adapted with permission from Popp et al. 2019

Stimulation was delivered through a multi-electrode configuration using the Ag/AgCl-ring electrodes that were already attached to the patient’s head. Using EEG electrodes yields a more focal stimulation and allowed us to customize spatial distribution of stimulation (Minhas et al. 2010). Stimulation electrodes were selected based on task-specific P300 topography (Fig. 4a, b) and finite element model (FEM) simulations of current flow (Fig. 4c, d, figure adapted from Popp et al. 2019). The presented FEM simulation was performed on an MNI standard brain and results in an electrical field strength of ~ 0.1 V/m in parietal and temporal cortices, which matches with the pattern of P300 generators (Bledowski et al. 2004). The main goal of the simulation was to ensure that the stimulated brain areas coincide with the targeted areas. The reader is referred to Popp et al. (2019) for the detailed description of the electrode selection procedure. Stimulation electrodes at EEG locations C3, C4, CP3, CP4, P3, and P4 were connected to the anode of our stimulator. Stimulation electrodes at EEG locations T7, T8, TP7, TP8, P7, and P8 were connected to the cathode of our stimulator. In order to achieve a relatively equal current distribution across the six electrodes per output, we assured that the electrode impedance was below 10 kOhm.

### Data Analysis

#### Online Analysis

The first part of the analysis was carried out during the experiment, in between the pre and during condition. It was performed in order to determine the individual stimulation parameters, namely the stimulation frequency and the peak latency of the stimulation function (see previous paragraph). A high amount of noise impeded the data collected in this ADHD sample. In order to increase the precision of parameter estimation, the time window for P300 latency estimation was changed from [0–900 ms] to [300–600 ms] and a single trial rejection routine was implemented in the online analysis (only), after patient 5. It included the visual inspection of target trials and the rejection of those that showed eye blinks (large deflection, uniform on all channels) in the time interval between 0 to 1 s after stimulus onset. This resulted in a mean trial number of 86.39 ± 5.51 used for parameter estimation. However, ERP waveforms still displayed some noise.

#### Offline Analysis

Due to the noise, the following additional preprocessing steps were employed for offline analysis after the completion of the experiment: 8 Hz low pass filter, re-referencing to average reference and independent component analysis (ICA). For ICA, the experimenter visually inspected and removed all components identifiable as artifacts, e.g., cardiac rhythm related, muscular artifacts related to movement or components showing a large amount of high frequency noise (mean number of rejected components for pre: 2.89 ± 1.57 and post: 3.11 ± 2.00). In order to avoid the rejection of components that contribute to P300 generation, components were only excluded if the time course of the component did not appear to have P300 typical activity in it (activity time-locked to stimulus onset peaking within 300 to 600 ms time window). Additionally, after the end of preprocessing an ERP analysis of the rejected ICA components was conducted to ensure that no P300 related activity was excluded from statistical analysis. Furthermore, resolution of the time frequency analysis for investigation of event-related spectral perturbation was increased (frequency range: 0.8 to 10 Hz, frequency resolution: 0.25 Hz, time resolution: 0.014 s).

#### Outcome Variables

From the preprocessed data, the following outcome variables were extracted for primary analyses:

- P300 amplitude and latency: P300 amplitude was defined as the maximum value [µV] of the averaged ERP waveform at Pz electrode in 300 to 600 ms time window after stimulus onset per condition and patient. P300 latency was its corresponding time point relative to stimulus onset [ms].
- Reaction time mean (RT-M) and reaction time variability (RT-V): reaction time (RT) was calculated based on EEG triggers, by subtracting the latencies of button presses and respective target stimulus onset. Means and standard deviations over trials were computed, serving as measures of RT-M and RT-V, respectively. Trials in which RT was longer than 1000 ms or less than 200 ms were judged invalid and were excluded from all analyses.
- Errors: two types of errors were investigated. All targets that were not followed by any button press were considered omission errors. Omission error trials were naturally excluded from reaction time analysis (mean number of trials: pre: 88.39 ± 7.37, during: 106.72 ± 17.75, post: 101.00 ± 6.49), while remaining in the EEG data analyses including P300 amplitude and latency estimation (mean number of trials: pre: 91.50 ± 0.71, post: 103.94 ± 0.24; see discussion in the Online Resource). A button press in response to a standard stimulus was classified as false alarm or commission error.

#### Testing Hypothesis 1: P300 Amplitude Modulation

The main research question of this study was whether P300 amplitude could be increased by the application of tACS in an ADHD patient group. As this hypothesis was directed, one-sided testing was employed. In order to compensate for variance across subjects, we computed the change of P300 amplitude from the pre condition to the post condition relative to the individual pre condition in %, i.e. (amplitude post − amplitude pre)/amplitude pre × 100. This amplitude change was then compared between the stimulation and the sham group. Due to the small sample size, Mann–Whitney test was employed (Shapiro–Wilk test, p > 0.05; see Table S1). All testing was performed at an alpha-level of 0.05.

#### Testing Hypothesis 2: Behavioral Effects

The secondary research question referred to potential behavioral changes in line with an amplitude modulation of the P300 component due to tACS. To this end, error rates, RT-M and RT-V were tested between experimental groups. The behavioral measures comprised three time points namely pre, during, and post intervention, as the experimental task was also performed during the application of tACS. We hypothesized a decrease in all the mentioned behavioral measures from pre intervention to during intervention and post intervention, which led to left-sided testing. Testing was performed on difference values (e.g., post condition − during condition) in % relative to the respective baseline condition (e.g., during for during-to-post comparison), except for errors, where this would have resulted in dividing by zero in some cases. As hypotheses were not directed for comparisons of during-to-post measures, two-sided tests were employed for the comparison of relative change during-to-post. As normality assumption was not met for at least one of each behavioral variable sets (Shapiro–Wilk test with p < 0.05, e.g. for pre condition RT-V, see Online Resource Table S1) Mann–Whitney tests were employed. Furthermore, due to the factor time comprising 3 time points (pre, during, and post), p-values were corrected for multiple comparisons. For that, FDR correction method by Benjamini and Hochberg (1995) was used, as implemented in the function *p.adjust* of the R software package.

#### Testing Underlying Assumptions

To further evaluate our results and test our assumptions several secondary analyses were conducted. The first subset of analyses served to validate the assumptions underlying our hypotheses. These were:

There is a relationship between P300 amplitude and behavioral measures relevant for ADHD symptomatology (subsections: P300 amplitude and behavior).

P300 amplitude is increased via the enhancement of its related ERO in the (stimulated) delta/theta frequency band as represented in the ERSP (subsections: delta/theta activity).

The second subset included additional analyses like of tACS side effects and the comparability of the experimental groups. All analyses employed Mann–Whitney tests, as used for the primary analyses. For relational assessments Spearman correlation was used.

##### P300 Amplitude and Behavior

To investigate the link between changes in P300 amplitude and behavioral measures as assumed by our hypotheses, a correlational matrix comparing relative changes of the outcome variables from pre-to-post was computed.

##### Delta/Theta Activity

In order to reveal potential event-related power changes in the stimulated frequency bands that we attribute to P300 activity, the relative change of event-related spectral perturbation (ERSP) was compared between groups. To this end, ERSP was analysed in the ± 3 Hz frequency and ± 150 ms time window around individual P300 latency and frequency used for stimulation. Subsequently, pre-to-post change, relative to the pre condition, of each patient’s maximum ERSP in the respective time- frequency window was determined, and groups were compared in a Mann–Whitney test comparison.

#### Additional Analyses

##### Parameter Estimation Error

Due to noise corruption and lower frequency resolution, there was an error in parameter estimation in the online analysis as evaluated based on the offline analysis. To consider its potential impact on the results, we investigated its relationship with all outcome variables in this study, for all patients as well as for the experimental groups separately. Here, the phase of the stimulation sinusoid at P300 Latency would be calculated for online parameters and offline parameters. Online parameters comprise P300 latency and frequency as estimated during measurement, whereas offline parameters comprise P300 latency and frequency after noise correction and using higher frequency resolution in time–frequency decomposition. The subtraction of the two phases resulted in the relative phase miss of stimulation which then served as quantification of parameter estimation error. Higher values correspond to a higher phase miss.

##### Group Comparability

Comparability of the experimental groups was tested for all baseline outcome measures as well as for the following demographic characteristics: age, gender, education [in years], medication [yes/no] and ADHD symptom severity as assessed via ADHS-SB questionnaire.

##### tACS Side-Effects

To administer tACS side-effects in this study, patients filled out a questionnaire after the measurement ended (as proposed by Brunoni et al. 2011). The questionnaire further included an item asking patients whether they believed to have been part of the stimulation or sham group (tick item including both groups). This procedure is used to foreclose possible confounds caused by the experimenter or different protocols and is in conformity with the current transcranial electrical stimulation protocol (Nitsche et al. 2008; Nitsche and Paulus 2007). Based on the questionnaire, a qualitative assessment was conducted of the subjective experience of tACS side-effects as well as the identifiability of group membership.

##### P300 Latency

For reasons of completeness, mean P300 latency was analyzed including its relation to reaction times in this sample. However, no tACS effect for P300 mean latency was expected under the given study design. Correlation was calculated for mean data across pre and post conditions.

## Results

### Findings Concerning Hypothesis 1: P300 Amplitude Modulation

**Fig. 5:**
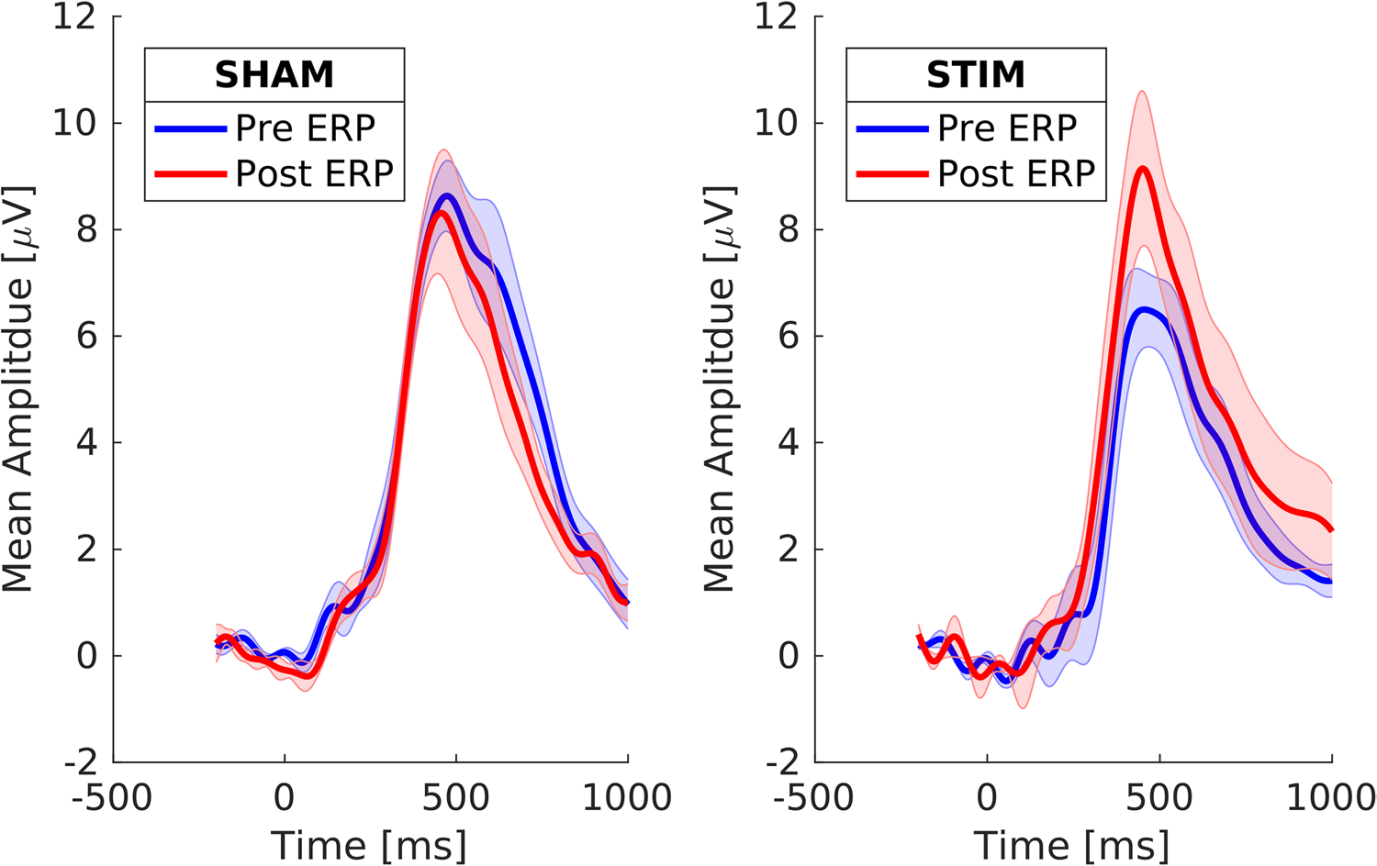
P300 modulation. Mean ERPs for all experimental groups and conditions. Shaded intervals display standard error of the mean at all time points. Left: in the sham group, ERPs were not significantly different between pre and post conditions. Right: in the stim group, the P300 amplitude was significantly larger in the post condition as compared to the pre condition. Note that statistics were not computed on absolute values but on relative amplitude change

Right-tailed Mann–Whitney test comparison testing for a higher increase of P300 amplitude, in % relative to pre condition, in the stimulation group (*M* = 26.49, *SD* = 33.90) than in the sham group (*M* = − 6.78, *SD* = 20.00) was significant at the chosen alpha level, *U* = 110, *p* = 0.016. Figure 5 displays the mean ERP at electrode Pz featuring P300 responses of stimulation and sham groups pre and post intervention. While the pre and post ERPs are very similar in the sham condition (left), there is a noticeable difference between the pre and post condition in the stimulation group (right).

As supported by the group comparisons regarding comparability, a group difference in P300 amplitude pre intervention was not significant (see “Group Comparability” section). Individual ERPs and pre-to-post data for statistical comparison can be found in the Online Resource.

**Fig. 6:**
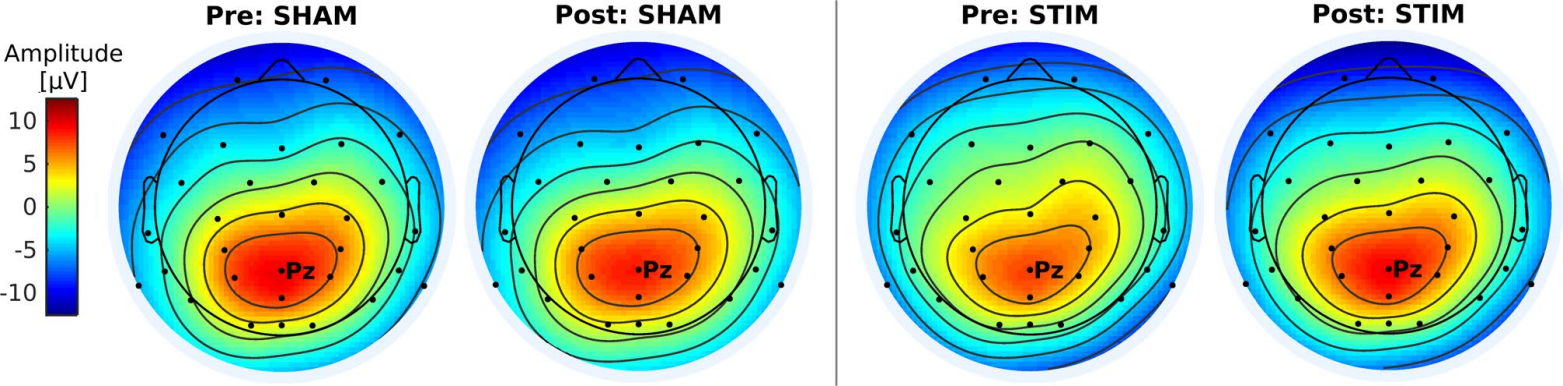
Average scalp topography at individual P300 latency (Pz) for group × condition

Accordingly, topographies at mean P300 latency (Fig. 6) showed P300-related parietal activity to increase in the stimulation group pre-to-post.

### Findings Concerning Hypothesis 2: Behavioral Effects

#### Reaction Time Mean and Variability

**Fig. 7:**
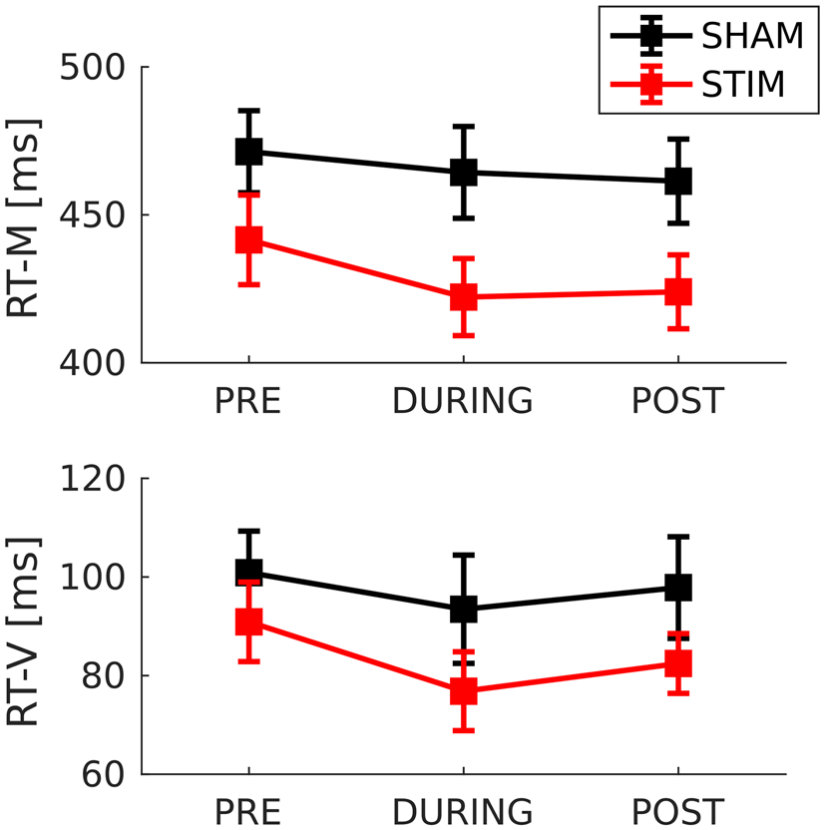
Reaction time mean and reaction time variability for group × condition

Comparisons for RT-M and RT-V were employed to test the hypothesized larger decrease (in % relative to baseline) in the stim group as compared to the sham group. None of the left-tailed Mann–Whitney tests yielded significant differences neither for pre-to-post comparison, nor for pre-to-during comparison. Moreover, two-tailed comparisons for during-to-post were not significant, neither for RT-M nor for RT-V. For detailed results the reader is referred to the Online Resource. Consequently, no significant tACS effect was found for the two reaction time measures in this sample, which are displayed in Fig. 7. Despite not being significant, a slightly larger decrease pre-to-post can be observed in the stim group for RT-M as well as RT-V. In general, stim patients showed higher RT performance than sham patients. However, group comparability analysis did not reveal significant differences for the pre condition (see “Group Comparability” section).

#### Error Rates

The chosen task was apparently easy for the patients, which can be inferred from the few omission errors and almost no false alarms that were made. Since the maximum number of false alarm type errors per condition was 4 (out of 96 trials) and overall mean 0.696 [absolute number], this type of error was disregarded for further analyses. Group comparison for pre-to-post revealed a larger decrease in absolute errors in the stim group (*M* = − 3.11, *SD* = 7.54) as compared to the sham group (*M* = 1.78, *SD* = 3.11). Accordingly, one-tailed Mann–Whitney test comparison was significant, *U* = 62.50, FDR corrected *p* = 0.027. The pre-to-during group comparison (*M*_*Stim*_ = − 3.22, *SD*_*Stim*_ = 7.22, *M*_*Sham*_ = 1.89, *SD*_*Sham*_ = 3.79), *U* = 63.50, FDR corrected *p* = 0.027, was equally significant. Both comparisons for omission errors showed descriptively into the expected direction, with best performance rates during tACS stimulation. Two-tailed comparison of during-to-post revealed an equal, low performance change in both groups (*M*_*Stim*_ = *M*_*sham*_ = 0.11, *SD*_*Stim*_ = 1.27, *SD*_*Sham*_ = 3.30), *U* = 77.00, *p* = 0.468. Stim patients maintained their higher performance level, while sham patients continued making more errors than in the pre condition.

### Findings Concerning Underlying Assumptions

#### P300 Amplitude and Behavior

The correlational matrix comparing relative changes of the outcome variables can be found in Table 1. Significant correlations were found for P300 amplitude and omissions (pre-to-post as well as pre-to-during with the pre-to-during > pre-to-post) and for RT-M and RT-V.

**Table 1:** Relationship between P300 amplitude and behavioral measures

		P3amp Pre-to-post	RT-M Pre-to-post	RT-M Pre-to-during	RT-V Pre-to-post	RT-V Pre-to-during	Omissions Pre-to-post	Omissions Pre-to-during
P3amp	Correlation	1	− 0.150	− 0.253	− 0.265	− 0.457	− 0.581*	− 0.673**
Pre-to-post	Sig. (2-tailed)	0	0.553	0.311	0.288	0.057	0.011	0.002
RT-M	Correlation		1	0.876***	0.633**	0.281	0.241	0.114
Pre-to-post	Sig. (2-tailed)		0	0.000	0.0048	0.257	0.335	0.651
RT-M	Correlation			1	0.612**	0.482*	0.123	0.026
Pre-to-during	Sig. (2-tailed)			0	0.007	0.043	0.628	0.918
RT-V	Correlation				1	0.311	0.139	0.196
Pre-to-post	Sig. (2-tailed)				0	0.210	0.583	0.435
RT-V	Correlation					1	0.2633	0.383
Pre-to-during	Sig. (2-tailed)					0	0.291	0.117
Omissions	Correlation						1	0.808***
Pre-to-post	Sig. (2-tailed)						0	0.000
Omissions	Correlation							1
Pre-to-during	Sig. (2-tailed)							0
	N	18	18	18	18	18	18	18

“Pre-to-post “ = (post – pre)/pre*100; “ pre-to-during “ = (during – pre)/pre*100

“RT-M”: reaction time mean, “-V”: variability. “P3amp”: P300 amplitude. “Sig.”: Significance Testing

*Correlation is significant at the 0.05 level (2-tailed). Correlation = Spearman Correlation

**Correlation is significant at the 0.01 level (2-tailed)

***Correlation is significant at the 0.001 level (2-tailed)

#### Delta/Theta Activity

**Fig. 8:**
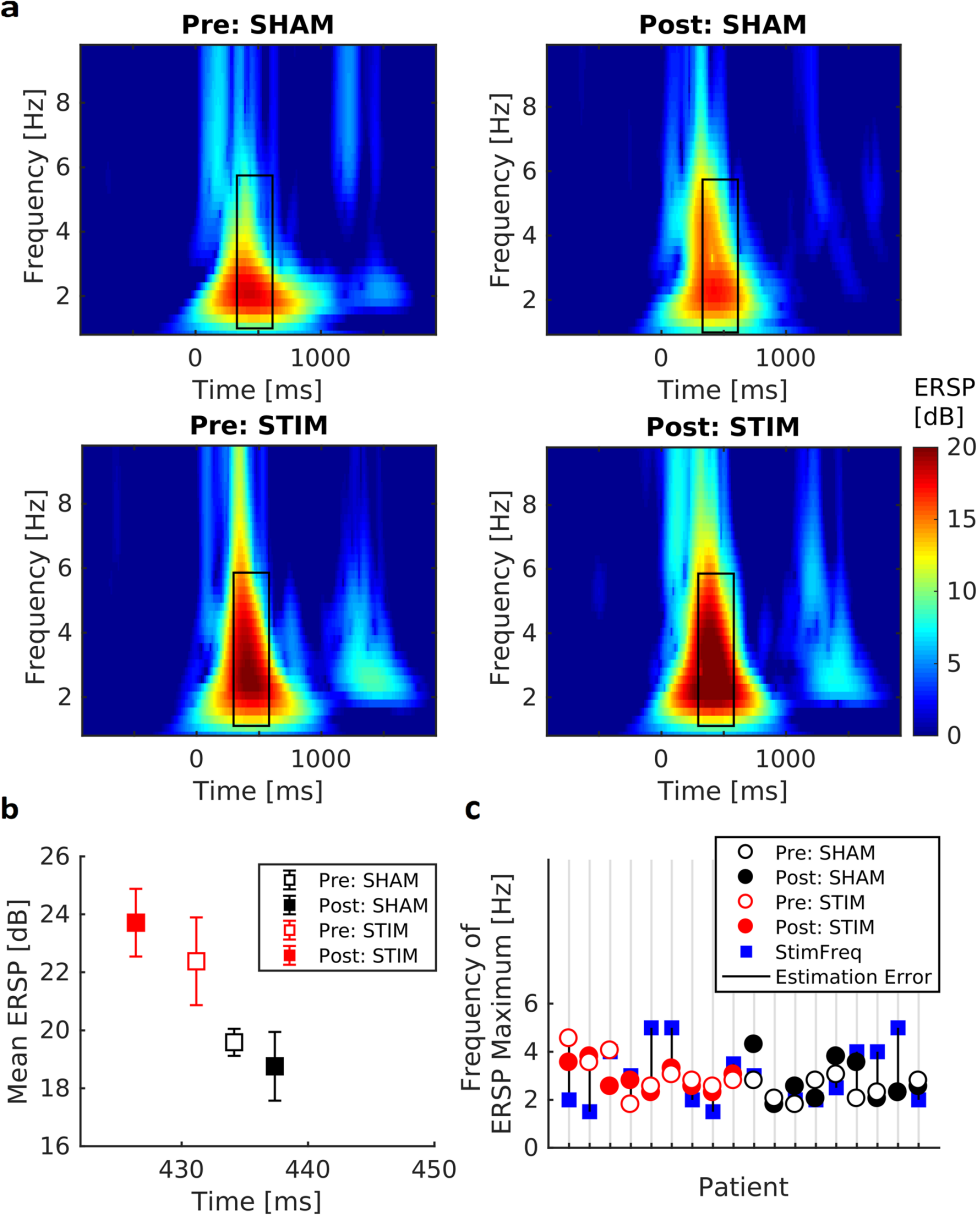
Event-related Spectral Perturbation. **a** Mean ERSP for group × condition. Black rectangle marks window around stimulation latency and frequency that was used for ERSP maximum detection for statistical comparison. **b** ERSP maximum mean and standard error for group × condition. ERSP maximum latency displayed on horizontal axis. **c** Frequency at individual ERSP maximum for group × condition. Used stimulation frequency (frequency of online pre condition ERSP maximum) marked in blue. Stimulation estimation error due to noise and low time–frequency resolution in the online analysis depicted as difference between frequencies of ERSP maxima online (“StimFreq”) and offline (“Pre:”)

Figure 8 displays mean ERSP in the employed two × two design. Black rectangles indicate respective mean of chosen time–frequency windows around online P300 latency and frequency that were used for offline ERSP maxima detection. On average, the windows capture ERSP maxima well: the strongest activity appeared in the estimated P300 time and frequency window. Furthermore, overall pattern of activity matched expected P300 pattern and showed similarity between the groups. However, overall mean intensity and frequency appeared to be lower in the sham group. The one-tailed Mann–Whitney test comparison testing our assumption of a relative increase of individual ERSP maxima pre-to-post in the stimulation group as compared to sham was not significant (*M*_*Stim*_ = 8.22, *SD*_*Stim*_ = 15.56, *M*_*Sham*_ = − 4.44, *SD*_*Sham*_ = 15.04), *U* = 1.755, *p* = 0.057. Descriptively however, stim patients showed an increase of ERSP peaks, while sham patients’ ERSP peaks decreased (Fig. 8b). Latency of ERSP peaks as well as frequency can be observed in Fig. 8 (horizontal axis in b and vertical axis in c, respectively).

### Findings concerning Additional Analyses

#### Parameter Estimation Error

Figure 8c shows frequency estimation errors in this study. There was a significant positive relationship between correct parameter estimation and omission error decrease: stim patients whose online P300 latency and frequency were estimated similarly in the offline analysis (after noise-correction and with higher frequency resolution in time–frequency analysis) showed a stronger performance increase in omission type errors. The relationship was not significant in the sham group. However, correct parameter estimation correlated significantly with RT-V pre-to-post decrease and estimation error in the sham group. Statistical results can be found in Table S3 of the Online Resource.

#### Group Comparability

The experimental groups did not reveal significant differences with regards to age, sex, medication, education or symptom severity as measured by ADHS-SB. Furthermore, none of the chosen primary outcome measures showed significant group differences pre intervention. An unexpected P300 amplitude group difference in the pre condition (Fig. 5 and Online Resource), (*M*_*Stim*_ = 7.65, *SD*_*Stim*_ = 3.57, *M*_*Sham*_ = 9.51, *SD*_*Sham*_ = 2.42) was not significant at the chosen alpha level, *U* = 68, *p* = 0.136. However, pre condition time–frequency characteristics (Fig. 8), (*M*_*Stim*_ = 22.38, *SD*_*Stim*_ = 4.55, *M*_*Sham*_ = 19.59, *SD*_*Sham*_ = 1.40), did differ significantly, *U* = 113, *p* = 0.014. Finally, performance levels in reaction time measures showed a group difference (Fig. 7), while not being significant for the pre condition.

The reader is referred to the Online Resource for statistical results of all experimental group comparisons (Table S2), as well as for the results regarding tACS Side Effects, and P300 Latency.

## Discussion

This paper introduces tACS as a possible candidate to influence the altered P300 component in ADHD.

As it is a pilot study with low sample size, results are of a preliminary nature only.

### Interpretation of Results

#### Hypothesis 1: P300 Amplitude Modulation

The most prominent finding of this study is the significant enhancement of P300 amplitude in the stimulation group as compared to sham. In line with our primary research hypothesis, the mean P300 amplitude of patients who received tACS stimulation within delta/theta frequency range was significantly increased after stimulation. Since patients in the sham condition did not show this effect, the stimulation group’s increase can be attributed to tACS stimulation. We conclude that tACS has potential to be used as a tool to manipulate altered P300 amplitudes in ADHD. However, this potential should be explored in further studies with larger sample sizes.

#### Hypothesis 2: Behavioral Effects

From a clinical perspective, behavioral benefits are a necessary outcome. In our sample, tACS induced a decrease in omission type errors. Regarding RT mean and variability, there was no significant effect indicating tACS-induced improvements. Descriptive changes in RT variability showed a decrease from pre-to-during, as observed in both experimental groups, and an increase from during-to-post. Those changes could be explained by learning and/or changes in alertness due to anticipation of stimulation. Potentially, an improvement of RT performance due to tACS could have been masked by effects as those mentioned. Regarding our second hypothesis, we conclude that tACS could induce behavioral improvements related to ADHD typical cognitive deficits producing omission type errors. Nevertheless, the behavioral outcome of a tACS intervention in ADHD requires further investigation.

#### Underlying Assumptions

Our assumptions found support in this data set. In line with our first underlying assumption, this data set indicated a relationship between the increase in P300 amplitude and a performance increase relevant for ADHD symptomatology: when P300 amplitude was increased after intervention, omission type errors decreased. Regarding our second underlying assumption, tACS induced changes in event-related spectral perturbation (ERSP) did not reach significance (*p* = 0.057). At a descriptive level, however, stimulated patients showed the expected increase in ERSP around the stimulation frequency, while sham patients showed a decrease.

#### Additional Analyses

The experimental groups in this study were comparable with regards to age, gender, ethnicity, education and severity of ADHD symptomatology. Statistical testing further did not indicate baseline group differences in P300 amplitude. However, we found a significantly larger maximum ERSP during the pre condition in the stim group as compared to the sham group. This could have influenced our results negatively. Finally, patients did not report significant discomfort with the stimulation, nor could they tell which experimental group they belonged to (see tACS Side Effects in the Online Resource). This indicates safety and applicability of tACS in the context of ADHD.

### Evaluation of Methodology

The data recorded in this ADHD sample featured a high amount of noise that caused challenges to data analysis. It resulted in imprecision of individual tACS parameter estimation after the pre condition. Increasing its precision is desirable in order to achieve the best possible stimulation outcome. In this study, reliability of P300 parameter determination for offline analysis could be increased by the following preprocessing: LP filtering with an 8 Hz cut-off, visual ICA artefact correction and re-referencing to average reference.

Main sources of noise (as apparent from ICA decomposition and visual inspection of the data) were related to eye blinks and facial muscle activity. Regarding hyperactivity being one of the core symptoms of ADHD, it seems reasonable to assume a relationship between ADHD characteristic behavior and the high amount of muscle related artefacts. This is supported by the notion that the data from a pilot study, which included a healthy sample, did not face the problem of increased noise (Popp et al. 2019). It is further affirmed by examples from the literature: for example, Fried et al. (2014) report increased rates of average microsaccade and blinks in ADHD patients (the authors even advocate for using the altered ocular activity as a marker in the differential diagnosis of ADHD). The larger blink rates and microsaccades in ADHD reported by Fried et al. were especially larger in the time interval around stimulus onset. The same observation was made in this study, even though the patients were instructed not to blink during stimulus presentation. Ochoa and Polich (2000) investigate the effects of instructions to suppress eye blinking on the P300 component. They find the visual oddball P300 amplitude to be smaller and its peak latency to be longer in the “do not blink” condition. ADHD patients often deal with difficulties to inhibit motor responses (Schachar et al. 2004). Therefore, instructing them to refrain from blinking might have caused problems in this study. In addition to noise in the data, low frequency resolution of the time–frequency decomposition in the online analysis (0.5 Hz) might have impeded the precision of stimulation frequency choice in this study. The relationship between better online parameter estimation and omission error improvement found in the stimulation group might indicate further potential of tACS to influence this behavioral outcome.

### General Discussion

Despite the abovementioned limitations, the overall study design proved to be feasible to investigate tACS-induced changes in P300 amplitude and behavioral changes relevant for ADHD symptomatology. Furthermore, the fact that P300 amplitude as well as spectral perturbation at individual P300 frequency increased pre-to-post stimulation indicates that tACS could potentially modulate EROs and related ERP components. However, further studies including bigger sample sizes and healthy controls are necessary to support these results. An interesting extension of our study design, which was based on mean P300 responses, would be to investigate tACS effects on single trial P300 activity. In the clinical context, this would allow to individually tailor tACS stimulation better accounting for interindividual differences in P300 latency variability. Additionally, single trial analyses would help to understand tACS effects as well as P300 generating mechanisms better: the increase in P300 amplitude found in our study could have resulted from either increases in single trial P300 amplitudes or decreases in single trial P300 latency via entrainment. This ambiguity could be resolved using single trial analyses. In line with the two different possible types of interpretation of our results, we further reference the reader to a related on-going debate regarding ERP generating mechanisms, namely the *evoked power* versus *phase reset model*, and suggest tACS as well as our specific study design extended by a single trial assessment to help elucidating the origin of ERPs. Finally, missing support for the manipulation of RT mean in this study could be explained by the same argument made regarding P300 latency: in this study, stimulation was delivered time-matched to mean P300 latency. Given the relationship between P300 latency and RT, this might also have set a constant time lag between stimulation and reaction time, prohibiting modulation of RT mean. We encourage future studies to further investigate the relationship between P300 amplitude and RT-V modulation during tACS, as it could help reveal possible entrainment effects. Furthermore, relating stimulation, single trial P300 responses and respective behavioral data would allow a more direct investigation of the effects of tACS in ADHD.

### Closing Comments

In this pilot study, tACS was introduced as a possible candidate to influence the altered P300 component in ADHD. Interestingly, ADHD is not the only disorder related to P300 alterations. For example, schizophrenia, Alzheimer’s disease and major depression have also been linked to decreases in P300 amplitude (Bramon 2004; Kaustio et al. 2002; Lee et al. 2013). Therefore, tACS might offer similar potential for other disorders. We reference the reader to recent studies investigating tACS effects in such disorders (Ahn et al. 2019; Alexander et al. 2019; Mellin et al. 2018). Furthermore, this study presented first support that tACS could be used to increase the amplitude of the P300 using its respective EROs as targets. We further suggest our study design to be extended by single trial assessment to shed light on the mechanisms of tACS-induced changes and the generation of ERP components including P300.

## Electronic supplementary material

Below is the link to the electronic supplementary material.

Electronic supplementary material 1 (PDF 970 kb)
