# Supplementary material for: Transcranial Alternating Current Stimulation (tACS) as a Tool to Modulate P300 Amplitude in Attention Deficit Hyperactivity Disorder (ADHD): Preliminary Findings
Source: Brain Topogr. 2020 Jan 23;33(2):191–207. doi: 10.1007/s10548-020-00752-x (PMC7066286; doi:10.1007/s10548-020-00752-x)
Supplement: Supplementary file 1 — Electronic supplementary material 1 (PDF 970 kb) [file 10548_2020_752_MOESM1_ESM.pdf]

*Brain Topography*

# Transcranial Alternating Current Stimulation (tACS) as a Tool to Modulate P300 Amplitude in Attention Deficit Hyperactivity Disorder (ADHD): Preliminary Findings

Dallmer-Zerbe I, Popp F, Lam AP, Philipsen A, Herrmann CS*

*** Corresponding Author:**

Experimental Psychology Lab, Department of Psychology, European Medical School, Cluster for Excellence "Hearing for All", Carl von Ossietzky University Oldenburg, Germany.

Email: christoph.herrmann@uni-oldenburg.de

## **Figure S1** P300 Amplitude Change

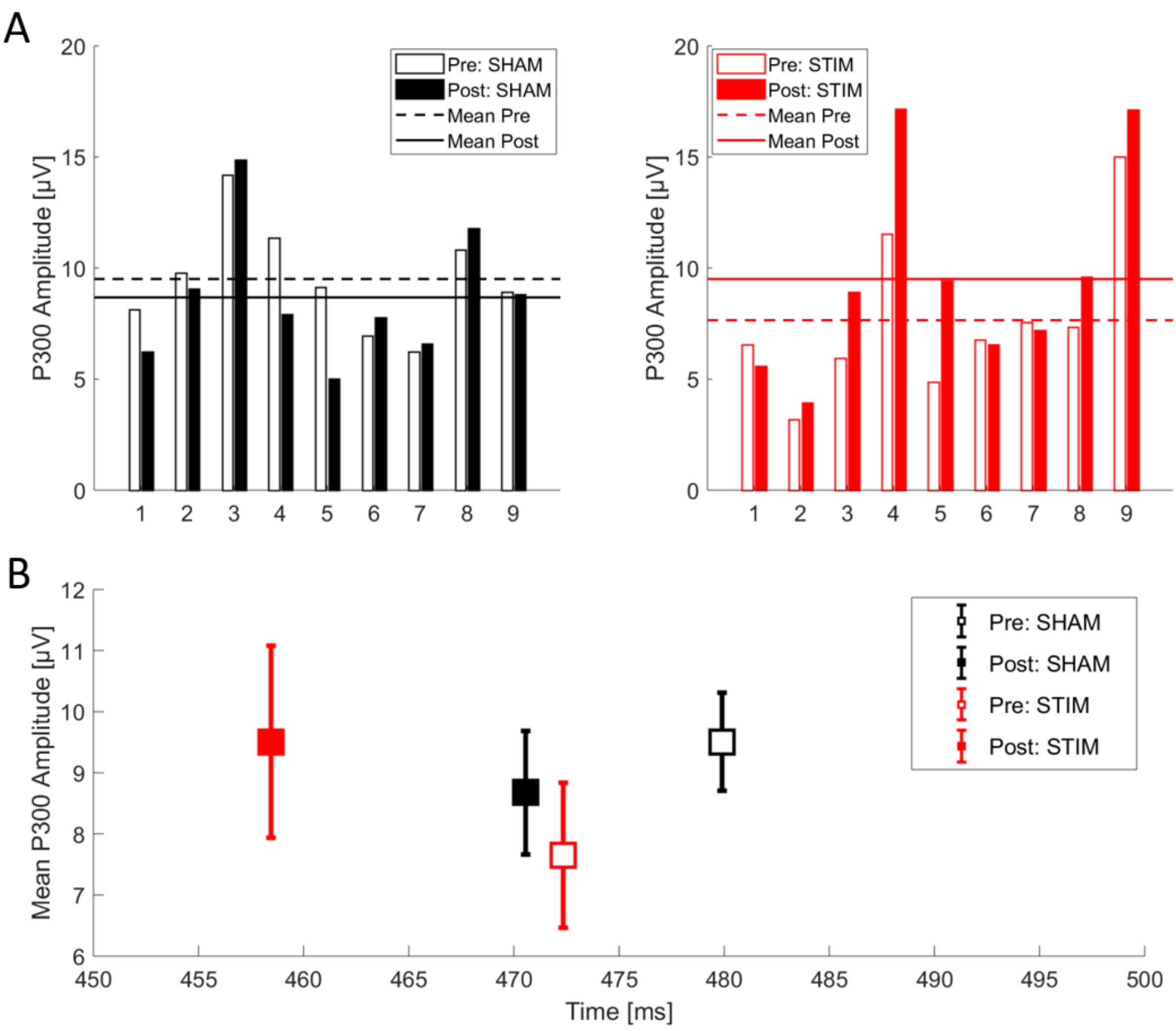


(A) Individual mean P300 amplitudes pre and post intervention as used for statistical testing. Left, sham group. Right, stimulation group. (B) P300 amplitude mean and standard error for group x condition. Respective P300 mean latency displayed on horizontal axis. Note that statistical testing of Hypothesis 1 was not computed on absolute values but on relative P300 amplitude change.

**Figure S2** Individual ERPs

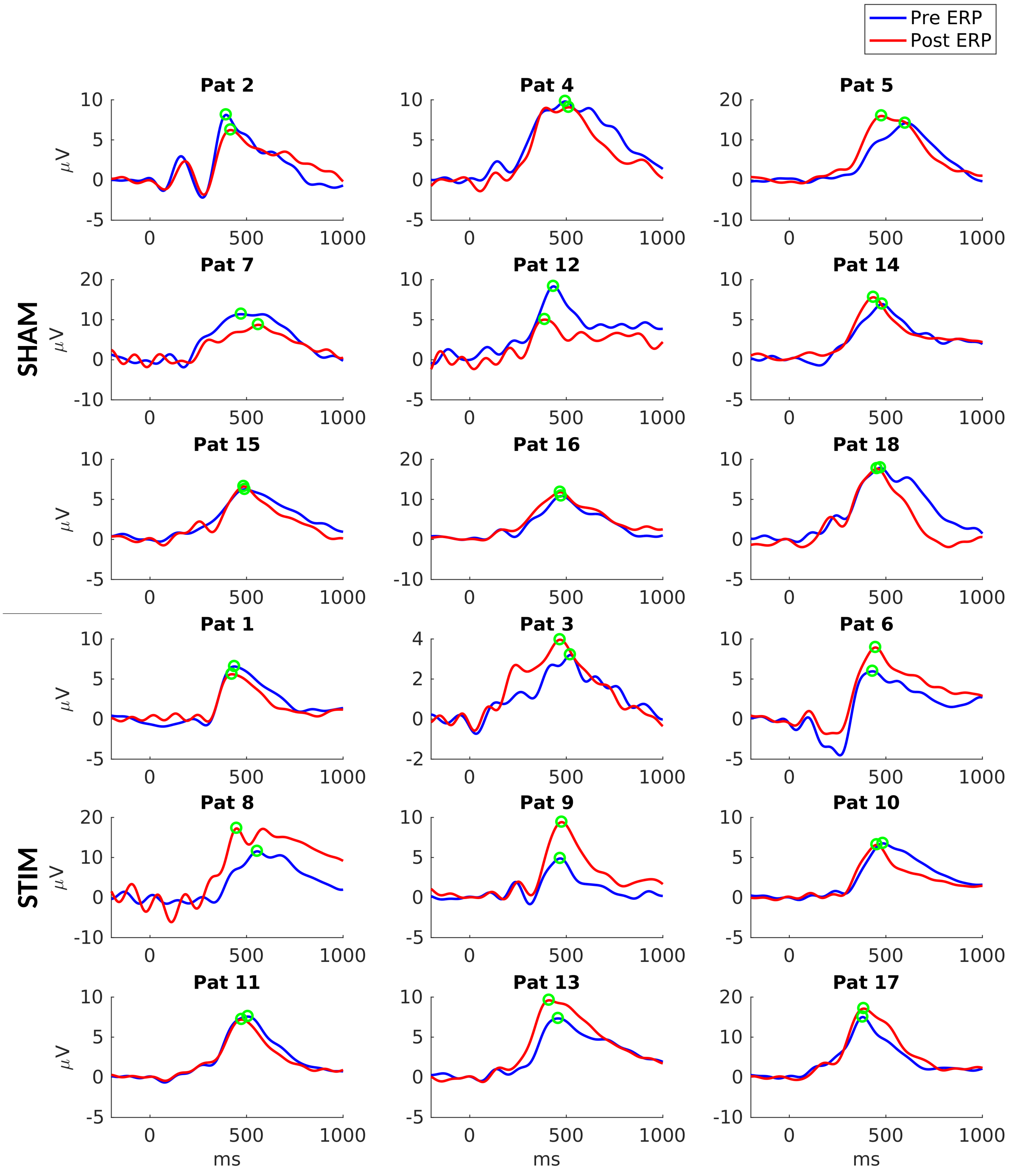


Green circles mark individual P300 amplitudes pre and post intervention.

# Outcome Variables: Trial Numbers

Two different types of trials were excluded from the analysis. Firstly, trials in which the response time exceeded a time window of 200 to 1000 ms. These trials were excluded from behavioral as well as EEG analysis. This concerns only two trials within the whole dataset. Secondly, trials were excluded when no response was given at all within the trial, so-called "omission errors". These trials were excluded only from the behavioral analysis, but remained in the EEG analysis. This approach could have altered the EEG results in an unfavorable manner. We argue that included omission error trials in the estimation of P300 amplitude and ERSP effects could have led to an underestimation of stimulation effects, as in the omission error trials small or no P300 component are expected. However, due to the low percentage of omission errors that occurred in all experimental conditions, we do not expect that our results were influenced considerably.

In order to investigate the potential effect of this issue, we analyzed the relation of omission errors relative to the overall number of trials. This resulted in average in values <5 %: $M_{stim}$ = 3.15 %, $SD_{stim}$ = 5.25 %, $M_{sham}$ = 3.78 %, $SD_{sham}$ = 7.80 %, $M_{block1}$ = 4.04 %, $SD_{block1}$ = 8.11 %, $M_{block3}$ = 2.89 %, $SD_{block3}$ = 6.25 %. A 2x2 mixed factorial ANOVA with the within-subject factor *time* (pre/ post intervention) and the between-subject factor *group* (stim/sham) was computed to identify possible differences in numbers of excluded trails between conditions. No differences were found for the factor *time* $F_{1,16} = 0.624$, $p = 0.441$, $\eta^2 = 0.007$, the factor *group* $F_{1,16} = 0.041$, $p = 0.842$, $\eta^2 = 0.002$ and interaction of *time* and *group* $F_{1,16} = 3.099$, $p = 0.095$, $\eta^2 = 0.033$.

**Table S1** Shapiro-Wilk Test: Statistical Results

| | | | | |
|---|---|---|---|---|
| | **P3amp** Pre-to-post [%] | **P3amp** pre [µV] | **ERSP** Pre-to-post [%] | **ERSP** pre [dB] |
| **NORMALITY** | | | | |
| m | 9.85 | 8.58 | 1.89 | 20.98 |
| s | 31.97 | 3.11 | 16.21 | 3.57 |
| W | 0.93 | 0.96 | 0.98 | 0.94 |
| p | 0.20 | 0.68 | 0.95 | 0.23 |
| | **RT-M** pre-to-post [ms] | **RT-M** Pre-to-during [ms] | **RT-M** During-to-post [ms] | **RT-M** pre |
| **NORMALITY** | | | | |
| m | -2.69 | -2.63 | 0.02 | 456.44 |
| s | 8.32 | 8.44 | 4.50 | 44.94 |
| W | 0.95 | 0.88 | 0.94 | 0.97 |
| p | 0.48 | 0.026 | 0.22 | 0.733 |
| | **RT-V** pre-to-post [ms] | **RT-V** pre-to-during | **RT-V** during-to-post | **RT-V** pre |
| **NORMALITY** | | | | |
| m | -5.17 | -11.85 | 9.24 | 95.89 |
| s | 20.42 | 14.20 | 25.73 | 24.54 |
| W | 0.94 | 0.94 | 0.96 | 0.81 |
| p | 0.28 | 0.27 | 0.58 | 0.004 |
| | **Omissions** pre-to-post [abs] | **Omissions** Pre-to-during [abs] | **Omissions** During-to-post [abs] | **Omissions** pre [abs] |
| **NORMALITY** | | | | |
| m | -0.67 | -0.67 | 0.11 | 3.67 |
| s | 6.14 | 6.18 | 2.42 | 7.51 |
| W | 0.59 | 0.60 | 0.72 | 0.55 |
| p | 0.000 | 0.000 | 0.000 | 0.000 |
| | **P3lat** Pre-to-post [%] | **P3lat** pre [ms] | **Age** [years] | **ADHS-SB** [abs points] |
| **NORMALITY** | | | | |
| m | -3.69 | 476.11 | 30.90 | 30.33 |
| s | 9.30 | 52.40 | 10.33 | 8.44 |
| W | 0.95 | 0.95 | 0.91 | 0.97 |
| p | 0.41 | 0.41 | 0.08 | 0.81 |
| | | **Education** [years] | **Gender** [1=f,0=m] | **Medication** [1=yes,0=no] |
| | **NORMALITY** | | | |
| | m | 14.97 | 0.39 | 0.53 |
| | s | 2.39 | 0.50 | 0.51 |
| | W | 0.88 | 0.62 | 0.64 |
| | p | 0.03 | 0.00 | 0.00 |

| SIGNIFICANCE TESTING: |
|---|
| **RIGHT** |
| **BOTH** |
| **LEFT** |
| p < 0.05 |

# Statistical Results for Reaction Time Measures

Reaction Time Mean

One-tailed two-sample Mann-Whitney tests yielded no significant differences, neither for **pre-to-post** comparison ($M_{Stim}$ = -3.63, $SD_{Stim}$ = 7.26, $M_{Sham}$ = -1.75, $SD_{Sham}$ = 9.61, $U$ = 83.00, FDR corrected $p$ = .647), nor for **pre-to-during** comparison ($M_{Stim}$ = -4.17, $SD_{Stim}$ = 5.33, $M_{Sham}$ = -1.08, $SD_{Sham}$ = 10.84, $U$ = 83, FDR corrected $p$ = .647).

Two-tailed comparisons for **during-to-post** were not significant ($M_{Stim}$ = .50, $SD_{Stim}$ = 3.56, $M_{Sham}$ = -0.46, $SD_{Sham}$ = 5.47, $U$ = 91, FDR corrected $p$ = .670).

Reaction Time Variability

Also, standard deviation of reaction times neither revealed a significantly larger decrease from **pre-to-post** in the stim group ($M$ = -7.50, $SD$ = 17.76) than in the sham group ($M$ = -2.84, $SD$ = 23.63, $U$ = 86, FDR corrected $p$ = .534), nor from **pre-to-during** ($M_{Stim}$ = -15.32, $SD_{Stim}$ = 10.78, $M_{Sham}$ = -8.37, $SD_{Sham}$ = 16.88, $U$ = 76, FDR corrected $p$ = .534).

Two-tailed comparisons for **during-to-post** yielded no significant differences ($M_{Stim}$ = 10.65, $SD_{Stim}$ = 23.59, $M_{Sham}$ = 7.82, $SD_{Sham}$ = 29.08, $U$ = 94, FDR corrected $p$ = .468).

# Findings concerning Additional Analyses

**Table S2** Experimental Group Comparability

| | Sample characteristics | | | | |
|---|---|---|---|---|---|
| | **Age** [years] | **Education** [years] | **Gender** [f=1, m=0] | **ADHS-SB** [abs points] | **Medication** [yes=1, no=0] |
| $M_{stim}$ | 34.15 | 14.72 | 0.33 | 29.78 | 0.50 |
| $SD_{stim}$ | 11.77 | 2.46 | 0.50 | 6.28 | 0.53 |
| $M_{sham}$ | 27.65 | 15.22 | 0.44 | 30.89 | 0.56 |
| $SD_{sham}$ | 8.01 | 2.44 | 0.53 | 10.55 | 0.53 |
| $U^{+}$ | 99.00 | 80.00 | 81.00 | 81.50 | 70.00 |
| *p* | 0.258 | 0.639 | 1 | 0.747 | 1.000 |

| | Outcome variables baseline | | | | | |
|---|---|---|---|---|---|---|
| | **P3amp** pre [µV] | **RT-M** pre [ms] | **RT-V** pre [ms] | **Omissions** pre [abs] | **ERSP** pre [dB] | **P3lat** pre [ms] |
| $M_{stim}$ | 7.65 | 441.54 | 90.90 | 4.44 | 22.38 | 472.33 |
| $SD_{stim}$ | 3.57 | 45.43 | 24.18 | 8.14 | 4.55 | 52.71 |
| $M_{sham}$ | 9.51 | 471.34 | 100.87 | 2.89 | 19.59 | 480.00 |
| $SD_{sham}$ | 2.42 | 41.57 | 25.29 | 7.24 | 1.40 | 55.26 |
| $U^{+}$ | 68.00 | 70.00 | 71.00 | 94.50 | 113.00* | 82.00 |
| *p* | 0.136 | 0.190 | 0.222 | 0.421 | 0.014 | 0.796 |

$^{+}$ Mann-Whitney Test, *significant $p<0.05$

**tACS side effects.** None of the patients reported discomfort with stimulation or terminated the experiment before it ended. Patients stated they believed to have received stimulation in 50% of the cases. Among those who thought to belong to the sham group, 66.7% were part of the stimulation group and vice versa. Hence, patients could not tell consistently which group they belonged to. Questionnaire results further showed that 66.7% of the stim patients experienced a tingling sensation caused by the stimulation. Further side effects related to tACS stimulation were mild itching, heating or slight pain sensation.

**Parameter estimation error.**

**Table S3** Parameter Estimation Error

| | | P3amp Pre-to-post | RT-M pre-to-post | RT-M pre-to-during | RT-V pre-to-post | RT-V pre-to-during | Omissions pre-to-post | Omissions Pre-to-during |
|---|---|---|---|---|---|---|---|---|
| Stim Patients | | | | | | | | |
| **ParEstErr** | Correlation | -.333 | -.317 | -.083 | -.050 | .500 | 0.742* | .627 |
| | Sig. (2-tailed) | .385 | .410 | .843 | .912 | .178 | .028 | .077 |
| | N | 9 | 9 | 9 | 9 | 9 | 9 | 9 |
| Sham Patients | | **P3amp** Pre-to-post | **RT-M** pre-to-post | **RT-M** pre-to-during | **RT-V** pre-to-post | **RT-V** pre-to-during | **Omissions** pre-to-post | **Omissions** Pre-to-during |
| **ParEstErr** | Correlation | -.167 | .500 | .367 | 0.750* | .267 | .235 | .327 |
| | Sig. (2-tailed) | .678 | .178 | .336 | .025 | .493 | .545 | .385 |
| | N | 9 | 9 | 9 | 9 | 9 | 9 | 9 |
| ALL Patients | | **P3amp** Pre-to-post | **RT-M** pre-to-post | **RT-M** pre-to-during | **RT-V** pre-to-post | **RT-V** pre-to-during | **Omissions** pre-to-post | **Omissions** Pre-to-during |
| **ParEstErr** | Correlation | -.115 | .333 | .288 | .419 | .301 | .217 | .24 |
| | Sig. (2-tailed) | .651 | .178 | .246 | .084 | .226 | .388 | .338 |
| | N | 18 | 18 | 18 | 18 | 18 | 18 | 18 |

* significant p < 0.05 ; Correlation = Spearman Correlation

**P300 latency.** For P300 latency, no significant difference was found pre-to-post intervention ($M_{Stim}$ = -5.45, $SD_{Stim}$ = 7.21, $M_{Sham}$ = -1.93, $SD_{Sham}$ = 11.17), $t(16) = -0.79$, $p = .439$. Spearman Correlation between mean RT and P300 Latency showed indication of significance in this study, $r = .253$, $p = .310$.
